# Poisoning by Lead

**Published:** 1854-12

**Authors:** 


Poisoning by Lead. Iodide of Potassium.—In twenty-three cases of saturnine disease—including colic, neuralgia, arthralgia, wrist drop (four cases), and general paralysis (six cases)—the iodide of potassium has been used by Dr. Swift, as recommended by Melesens. Sixteen cases were cured; three so far relieved as to be able to resume their occupations; and four were gradually improving at the time the report was made. "In thirteen cases the urine was submitted to chimical analysis, and the investigation has established the fact, that the lead may be eliminated from the system by the iodide of potassium, and found in the urine. In no case was the lead detected before the administration of the remedy. The analyses were made by Prof. Outram, and the results of his experiments are perfectly reliable." In one case the lead was detected also in the saliva.—*N. Y. Med. Times.*

